# Establishment and evaluation of a transgenic mouse model of arthritis induced by overexpressing human tumor necrosis factor alpha

**DOI:** 10.1242/bio.016279

**Published:** 2016-03-14

**Authors:** Ge Li, Yu'e Wu, Huanhuan Jia, Lu Tang, Ren Huang, Yucai Peng, Yu Zhang

**Affiliations:** 1 Guangdong Laboratory Animals Monitoring Institute, Guangdong Provincial Key Laboratory of Laboratory Animals, Guangzhou, Guangdong 510663, China; 2 Livzon MabPharm Inc., No. 38 Chuangye North Rd., Jinwan, Zhuhai, Guangdong 519045, China

**Keywords:** Animal model, Transgenic, TNFα, Rheumatoid arthritis, Synovial hyperplasia, Drug evaluation

## Abstract

Tumor necrosis factor alpha (TNFα) plays a key role in the pathogenesis of rheumatoid arthritis (RA). Blockade of TNFα by monoclonal antibody has been widely used for the therapy of RA since the 1990s; however, its mechanism of efficacy, and potential safety concerns of the treatment are still not fully understood. This study sought to establish a transgenic arthritic mouse model by overexpressing human TNFα (hTNFα) and to apply this model as a means to evaluate therapeutic consequences of TNFα inhibitors. The transgenic mouse line (TgTC) with FVB background was generated by incorporating 3′-modified *hTNFα* gene sequences. A progressively erosive polyarthritis developed in the TgTC mice, with many characteristics observed in human rheumatoid arthritis, including polyarticular swelling, impairment of movement, synovial hyperplasia, and cartilage and bone erosion. Gene expression analysis demonstrated that hTNFα is not only expressed in hyperplastic synovial membrane, but also in tissues without lesions, including brain, lung and kidney. Treatment of the TgTC mice with anti-hTNFα monoclonal antibodies (mAb) significantly decreased the level of hTNFα in the diseased joint and effectively prevented development of arthritis in a dose-dependent response fashion. Our results indicated that the TgTC mice represent a genetic model which can be used to comprehensively investigate the pathogenesis and therapeutics of TNFα-related diseases.

## Abstract (precis)

**Summary:** We describe the establishment of a human TNFα transgenic arthritis mouse model with applications for understanding the role of TNFα in disease progression and developing therapeutic strategies.

## INTRODUCTION

Tumor necrosis factor alpha (TNFα) has a particularly important role in the cascade of pathogenic events in rheumatoid arthritis (McInnes and Schett, 2011). High level expression of TNFα in synovium induces joint inflammation and proliferation of fibroblast-like synoviocytes (FLSs). It further triggers cartilage destruction by inducing collagenase expression, inhibits proteoglycan synthesis by articular chondrocytes, and stimulates osteoclastogenesis and bone resorption (Li and Schwarz, 2003).

Since the early 1990s, TNFα has become a validated therapeutic target for the treatment of several autoimmune disorders including rheumatoid arthritis. Several anti-TNFα biologics were commercialized successfully with global sales exceeding USD 25 billion per year (Monaco et al., 2015). However, the mechanisms, particularly the downstream changes of the signal transduction cascades after TNFα neutralization behind the TNFα-blocking therapy remain unclear. Additionally, the cost of anti-TNFα therapy has become a burden to both the public and patients. Therefore, understanding function of TNFα in autoimmune diseases and developing affordable, safe, and equally potent anti-TNFα analogues is still ongoing worldwide.

Due to spontaneous development of the symptom of rheumatoid arthritis, human TNFα (hTNFα) transgenic mice became the most successful transgenic model used to investigate the molecular mechanisms of the pathogenic process and evaluate the efficacy of novel therapeutic strategies for rheumatoid arthritis. Based on a previous report (Keffer et al., 1991), several transgenic mouse lines were established in the past 30 years by over-expressing human TNF-α, and developed an erosive polyarthritis, these models exhibited many characteristics observed in rheumatoid arthritis patients. However, because of intellectual property issues, these models are not easily accessible for development of anti-TNFα medicines in developing countries.

Here we generated an hTNFα transgenic mouse line TgTC carrying and expressing a 3′-modified human *TNFα* gene construct. The mice showed several typical symptoms of rheumatoid arthritis, including swelling of the joints, synovial hyperplasia and cartilage and bone erosion, etc. Anti-TNFα therapy can effectively suppress the pathogenesis of arthritis in TgTC mice. The results from this study indicate that this transgenic line is a valuable model for the study of TNFα role in disease progression and therapeutic areas.

## RESULTS

### Appearance of chronic inflammatory polyarthritis in TgTC mice

**Fig. 1.**
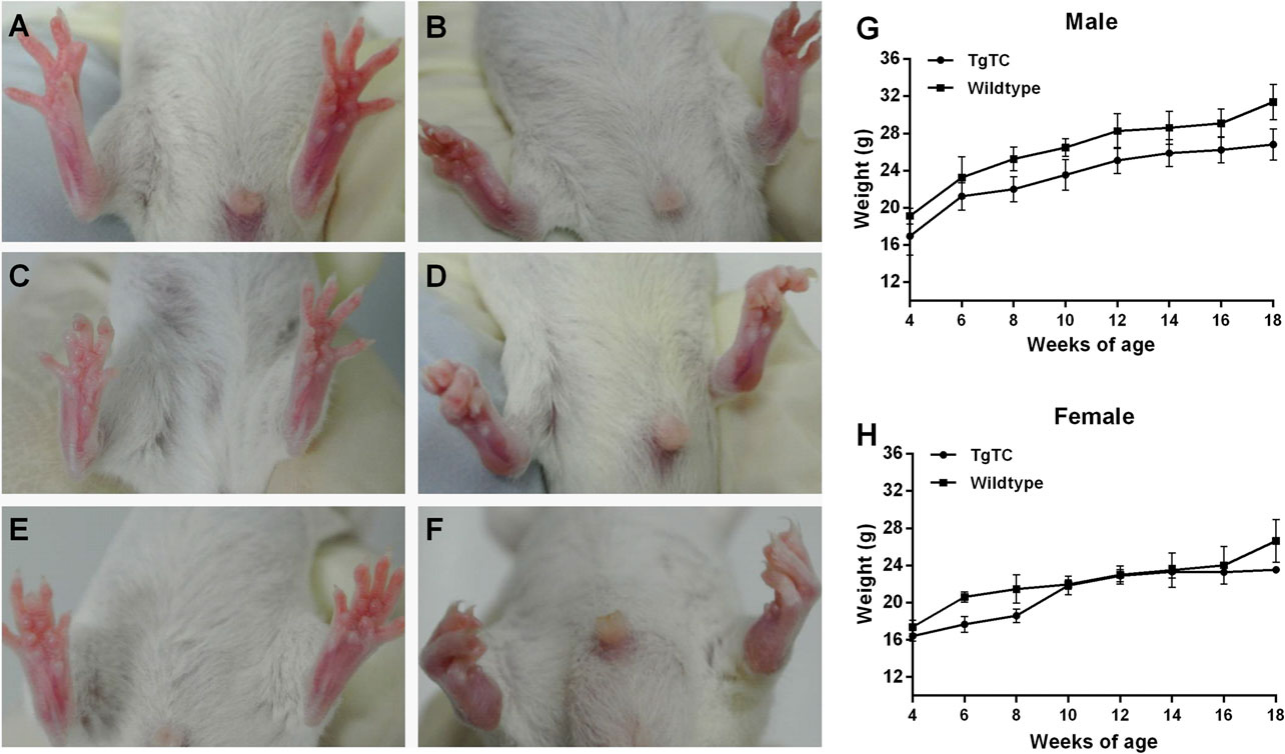
**Clinical characteristics of TgTC mice.** Comparing to the control (A, 3 weeks; C, 10 weeks; E, 20 weeks), the hind limbs of 3 week (B), 10 week (D) and 20 week (F) TgTC mice show progressive symptoms of arthritis. The weight of male (G) and female (H) TgTC mice did not appear to be significantly lighter than littermate controls (*n*>5). Data presented as mean±s.e.m.

The transgenic line of TgTC showed a 100% frequency with the macroscopic polyarthritis pathology, including swelling of the joints and impairment in movement, etc. The onset of the disease was at 2–3 weeks of age before the progeny were weaned. Comparing to wild-type mice (Fig. 1A), the transgenic mice showed a normal ability of movement, but ankle joints appeared minimal swelling and distortion (Fig. 1B). The symptom progressed to moderate disease at 9–10 weeks of age, with twisting of hind paws, moderate swelling of joints and distortion (Fig. 1C, wild type; Fig. 1D, TgTC). At 18–20 weeks, these mice suffered serious polyarthritis symptoms, including loss of movement in their hind legs, severe joint stiffness, swelling and distortion (Fig. 1E, wild type; F, TgTC). However, the body weight of TgTC mice did not show significant differences from their littermate control (Fig. 1G, male; Fig. 1H, female).

### Histology of peripheral joints of TgTC mice

**Fig. 2.**
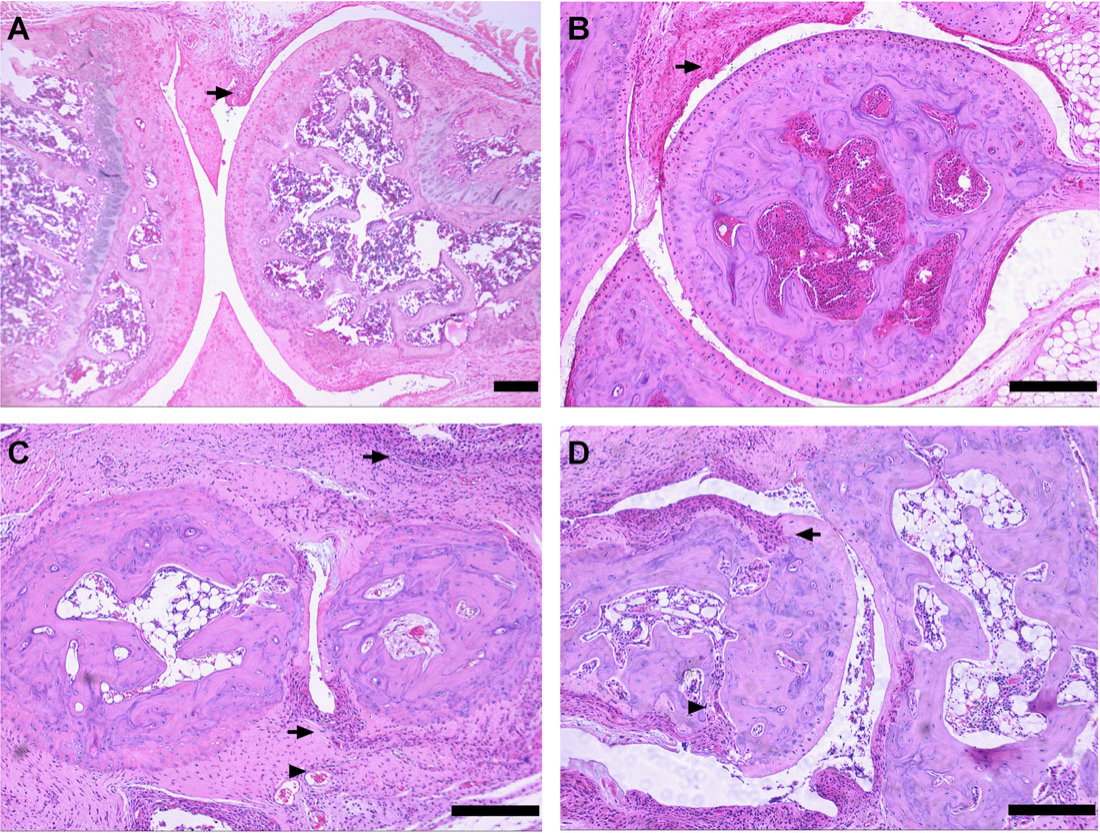
**Histology of peripheral joints showing the progression of arthritis at different developmental stages of TgTC mice.** Hyperplasic synovium (arrow) in the knee (A) and elbow (B) joint of TgTC mice at 3 weeks. Pannus formation (arrowhead) and fibrous tissue (arrow) in the ankle joint (C) of TgTC mice at 10 weeks. Cartilage (arrow) and bone (arrowhead) erosion in the ankle joint (D) of TgTC mice at 18 weeks. Scale bars=200 μm.

To verify histopathological changes in the animals with macroscopic signs of the disease, we performed histological analyses on peripheral joints from TgTC mice. Typical hyperplasia of the synovial membrane were the main feature found in nearly all joints examined at different developmental stages of TgTC mice. Analysis also revealed that synovial thickening was in accordance with macroscopic symptoms, and the onset of arthritis started at around 2–3 weeks of age (Fig. 2A,B). As the disease progressed, pannus formation and massive production of fibrous tissue were observed in suffered joints (Fig. 2C). In final, it resulted in massive cartilage and subchondral bone erosion was observed in the joints of transgenic animals (Fig. 2D), accompanied by impairment of moving capability. All these histological characteristics observed in TgTC mice were highly similar to symptoms of advanced human rheumatoid arthritis. Other than joints, no additional histological abnormalities were found in TgTC mice (data not show).

### Expression of the transgene in TgTC mice

**Fig. 3.**
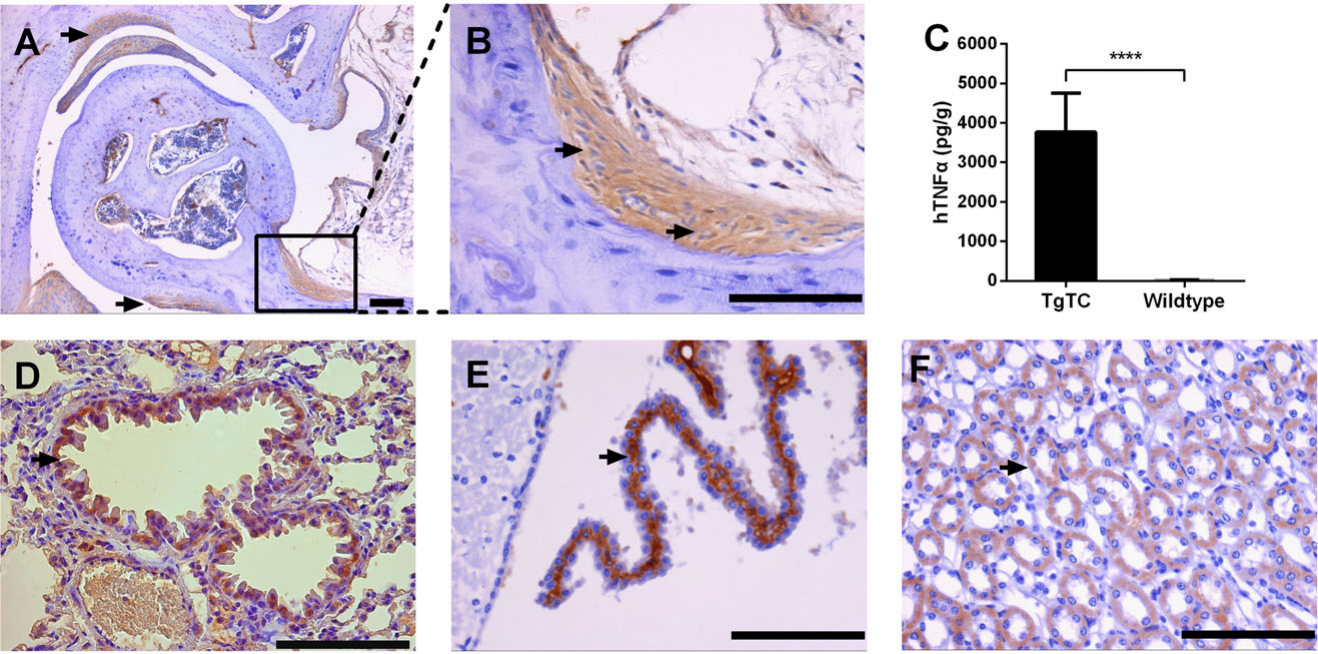
**Expression of hTNFα in the joint and other tissues of TgTC mice.** (A) Expression of hTNFα in hyperplastic synovium (arrow) in the elbow joint of TgTC mouse at 9 weeks of age. (B) Higher magnification of boxed synovium in panel A showed hTNFα was produced by fibroblast-like synovial cells (arrow). (C) hTNFα was significantly higher in synovial tissue of TgTC mice than that of wild-type (wild type, 11.65±7.774 pg/g; TgTC, 3771±346.7 pg/g). Data presented as mean±s.e.m. Asterisk indicates a significant difference between wild-type and TgTC. ****P<0.0001 (*t*-test), wild-type versus TgTC. (D-F) Expression of hTNFα in columnar epithelial cell (arrow) in bronchioli terminals (D), neurocytes (arrow) in cerebral cortex (E) and renal tubular epithelial cells (arrow) in medulla kidney (F) of TgTC mouse at 9 weeks of age. Scale bars=100 μm.

To investigate the relationship between the transgene and the disease of TgTC mice, we respectively utilized immunohistochemistry and cytometric bead array to analyze the expression profile of hTNFα in TgTC mice. In peripheral joints, significantly positive immunostaining was found in cells of synovial lining layer and deeper synovial tissue around the eroded cartilage (Fig. 3A). The majority of cells positive for hTNFα expression were elongated and spindle shaped, which were characteristics of a fibroblast-like morphology (Fig. 3B), suggesting that synovial fibroblasts express the human transgene. Quantification analysis also indicated that protein levels of hTNFα in synovial tissue of TgTC mice were significantly increased (Fig. 3C, wild type, 11.65±7.774 pg/g; TgTC, 3771±346.7 pg/g; *P*<0.0001). In addition, we found that hTNFα was also expressed in other tissues without histological abnormalities. These analyzed tissues included ciliated columnar epithelial cells in bronchioli terminals (Fig. 3D), neurocytes in the cerebral cortex (Fig. 3E), and renal tubular epithelial cells in medulla kidney (Fig. 3F).

### Suppression of arthritis symptoms in TgTC mice by administrating anti-hTNFα monoclonal antibodies

**Fig. 4.**
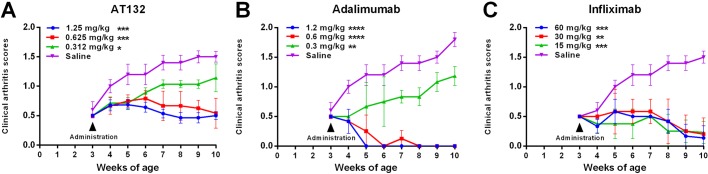
**Clinical scores of arthritis symptoms in TgTC mice after treatment with anti-hTNFα monoclonal antibodies.** Different anti-hTNFα monoclonal antibodies dissolved in saline were intraperitoneally administered to TgTC mice weekly from three to ten weeks, with saline-treated TgTC mice serving as control. (A) AT132 (1.25, 0.625, 0.312 mg/kg), (B) Adalimumab (1.2, 0.6, 0.3 mg/kg) and (C) Infliximab (60, 30, 15 mg/kg). Data presented as mean±s.e.m. Asterisk indicates a significant difference between saline and anti-hTNFα monoclonal antibody treated. *P<0.05, **P<0.01, ***P<0.001 and ****P<0.0001 (*t*-test), saline versus anti-hTNFα monoclonal antibody treated.

To further demonstrate that the expression of transgene caused polyarthritis in TgTC mice and evaluate whether TgTC mice were a potential animal model useful for designing protocols aiming at prevention or therapy in TNFα-related diseases, an anti-hTNFα monoclonal antibody, AT132, was intra-peritoneally administered to TgTC mice weekly from three to ten week age. Compared to saline-injected transgenic littermates which developed macroscopical symptoms of arthritis, AT132-treated TgTC mice showed significantly inhibited clinical score of arthritis (*P*<0.01). When drug concentration was greater than 0.625 mg/kg, dose-dependent response of AT132 was well-exhibited in the animal model (Fig. 4A). Meanwhile, in accordance with the same administrated procedure as AT132, two commercially available anti-hTNFα monoclonal antibodies, Adalimumab at 12, 6, 3 mg/kg (Fig. 4B) and Infliximab at 60, 30, 15 mg/kg (Fig. 4C), were also able to inhibit development of the symptom in TgTC mice.

**Fig. 5.**
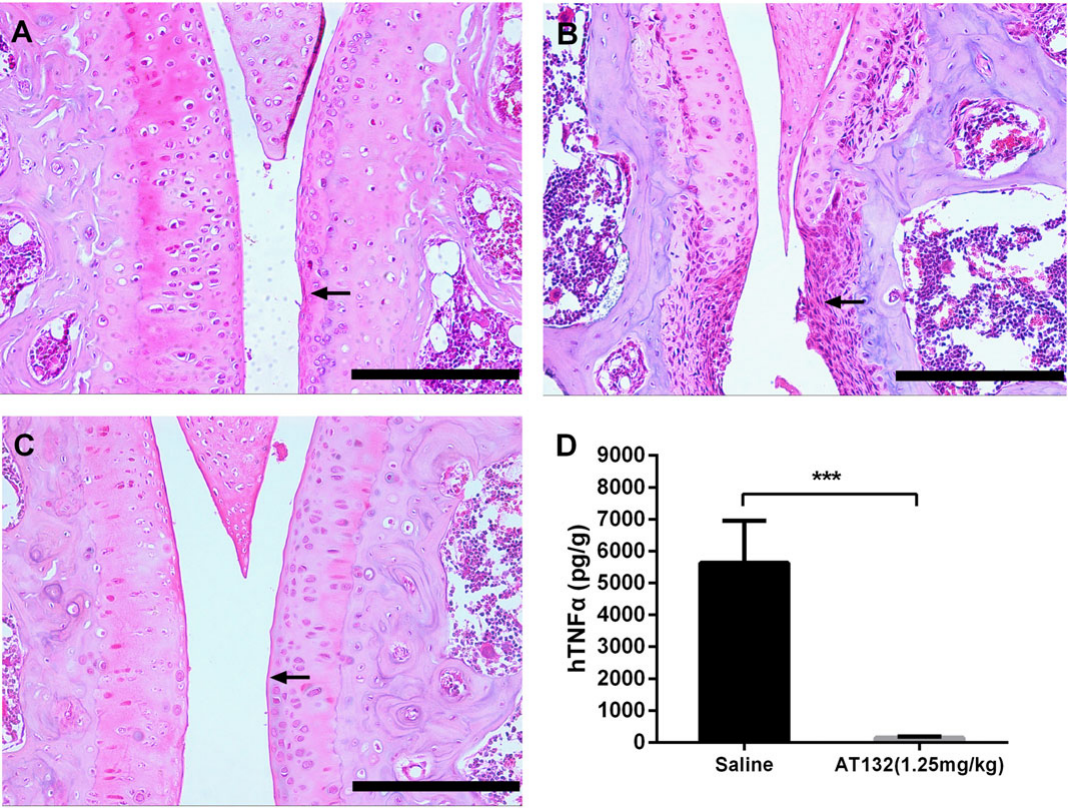
**Administration of AT132 can effectively suppress the development of arthritis in TgTC mice.** Sections of the knee joint at 8 weeks of age: wild-type mice (A), TgTC mice treated by saline weekly (B) and TgTC mice treated by AT132 at 1.25 mg/kg weekly (C) are shown. Thickening of the synovial layer (arrow) is completely suppressed in the antibody-treated transgenic mice. Treatment by AT132 at 1.25 mg/kg weekly can inhibit hTNFα production in synovial tissue of TgTC mice (D) (TgTC, 5635±585.7 pg/g; TgTC+AT132 1.25 mg/kg, 151.6±14.92 pg/g). Data presented as mean±s.e.m. Asterisk indicates a significant difference between saline- and AT132-treated samples. ***P<0.001 (*t*-test), Scale bars=200 μm.

Histological analysis of joints was performed in wild-type, AT132- (1.25 mg/kg) and saline-treated TgTC mice sacrificed at 10 weeks of age, synovial thickening, cartilage fibrosis and bone erosion were found in different peripheral joints of saline-injected transgenic mice (Fig. 5B). By contrast, antibody-treated animals were normal in appearance at 10 weeks of age (Fig. 5C) in accordance with non-transgenic control animals sacrificed at the same age (Fig. 5A). In addition, we analyzed the level of hTNFα in AT132 and saline-treated TgTC mice, and found that the cytokine in synovial membrane of TgTC mice significantly decreased after AT132 therapy (Fig. 5D, saline-injected, 5635±585.7 pg/g; AT132-injected, 151.6±14.92 pg/g; *P*<0.001).

## DISCUSSION

In the present study, we established a progressive arthritis mouse model by endogenously expressing hTNFα transgene (Keffer et al., 1991; Williams et al., 1992; Hayward et al., 2007). The mouse line was validated through clinical evaluation, histopathological analysis, transgenic expression and response to anti-hTNFα treatment. We conclude that the model is suitable for research and development of drugs for TNFα-relative diseases.

To ensure stable expression of hTNFα *in vivo*, a modified *hTNFα* transgene was constructed, in which the endogenous 3′ UTR of *hTNFα*, thought to be required for the regulation of mRNA stability and translational efficiency (Kruys et al., 1989; Han et al., 1990), was replaced by that of human *β-globin* (Keffer et al., 1991). By microinjecting the transgenic construct into pronuclear zygotes of FVB mice, several transgenic lines with different levels of progressive arthritis were obtained. Comparable to the most extensively studied line Tg197 (Keffer et al., 1991), a transgenic line named TgTC was selected for this study. With respect to key clinical features, TgTC mice manifested the symptom of chronic inflammatory polyarthritis after weaning, which is consistent with Tg197 as described (Keffer et al., 1991), but the onset of serious syndrome, such as complete loss of movement of the hind legs, happened around 18–20 weeks compared with 9–10 weeks for Tg197. Because disease progression of TgTC mice was comparatively slow, cachexia, considered as a hallmark in previously reported Tg-hTNFα lines using the same transgenic construct (Li and Schwarz, 2003; Hayward et al., 2007), was not as severe as other hTNFα transgenic lines before 18 week age; it is possible that weight loss would be shown when the observation period was further extended. On the other hand, random integration of multiple transgene copies to the mouse genome led to differential expression of hTNFα and influenced expression of other cellular genes, which potentially caused the differences of phenotypes in different transgenic lines.

Previous reports indicated that differentiation of major histocompatibility complex (MHC) in different inbred lines influenced the susceptibility of mice to collagen-induced arthritis (CIA) (Backlund et al., 2013; Holmdahl et al., 1988). For hTNFα transgenic mice model, the susceptible strain, DBA/1, exhibited more aggressive arthritis than those resistant strain, C57BL/6 (Butler et al., 1997). Our TgTC mice was developed from a resistant-strain FVB (Osman et al., 1999), therefore it should not be surprising to see different disease progression compared to other transgenic lines.

As a typical histopathological feature of rheumatoid arthritis, hyperplastic synovial membrane was easily observed in nearly all examined joints of TgTC mice in different development stages, and the severity of hyperplasia correlated with the deterioration in health of these mice. In addition, immunostaining and cytometric bead array analysis showed that majority of cells in synovial tissue express hTNFα; this is especially evident for fibroblast-like cells, which play a key role in erosive arthritis (Redlich et al., 2002; Lee and Weinblatt, 2001). Experimental evidence has suggested that fibroblasts within the synovial tissue can be activated by TNFα, leading to invasion and resorbing of cartilage and bone in RA (Sack et al., 1999). Furthermore, hyperplastic synovial tissue can be considered to provide a suitable microenvironment for osteoclastogenesis, since synovial fibroblasts and immune cells serve as potential nursing cells (Gravallese et al., 2000; Takayanagi et al., 1997), and express RANKL to induce differentiation of osteoclast directly involved in lesions of RA (Yang et al., 2014).

Though the transgene construct was randomly integrated to the genome, expression of hTNFα was restricted to a few organs, such as joint, lung, kidney and skin, in TgTC and other studied transgene lines (Hayward et al., 2007; Butler et al., 1997). However, transgene expression could not cause pathologic changes detectable in these tissues, except in the joints. Compared to other organs, we considered that the micro-environment of joints is relatively enclosed and static, which facilitate accumulation of synovium-produced hTNFα to pathological levels.

Tumor necrosis factor alpha is one of the main trigger of chronic inflammation in rheumatoid arthritis (McInnes and Schett, 2011). As a therapeutic strategy, the anti-TNFα biologics have revolutionized the treatment of TNFα related diseases (Taylor, 2010). Therefore, experimental arthritis models play an important role in the basic understanding of joint disease and in the development of effective anti-arthritic agents. In this study, by using the TgTC mice, we found that in the same treatment procedure, although effective doses of AT132, Adalimumab and Infliximab were different, administration of these anti-hTNFα mAbs could effectively inhibit progression of arthritis. Meanwhile, dose-dependent responses were well-demonstrated in the transgenic animal model when dosage gradient of antibodies was appropriately chosen. These results also suggested that arthritic phenotype of TgTC mice was stable and reliable. In addition, we analyzed the histopathological characteristic of joints after treating with AT132, we found synovial hyperplasia was effectively controlled in peripheral joints, when the dosage was greater than 0.625 mg/kg, the mAb significantly decreased the content of hTNFα in the diseased joint.

In summary, the progressive arthritis observed in TgTC mice is similar to the pathology in patients with RA. The blockade of hTNFα significantly reduces the signs and symptoms of RA in the animal model. Our study on TgTC mice demonstrates the usefulness of this mouse line as a model for study of TNFα-relative human diseases and anti-TNFα treatment.

## MATERIALS AND METHODS

### Animals

All of animal experiments in this study were approved and reviewed by Institutional Animal Care and Use Committee of the institute.

The TgTC mice were generated using a human *TNF/β-globin* (*TNF-globin*) recombinant gene construct, which contains a 2.8 kb fragment with the entire coding region and promoter of the *hTNFα* gene, fused to a 0.77 kb fragment with the 3′ untranslated region (UTR) and polyadenylation site of human *β-globin* replacing that of the *hTNFα* gene, as previously reported (Keffer et al., 1991). The fragment was then microinjected into pronuclei of FVB/J inbred strain fertilized eggs. Finally, the injected fertilized eggs were implanted into the oviduct of 8-week-old female pseudo-pregnant ICR mice. Transgenic lineages were established by back-crossing the transgenic founder individuals to the FVB/J inbred strain. The genotyping was performed by PCR to screen for transgenic animals as well as routine tail genotyping. The transgene specific PCR primers were: 5′-GAΑCTCCCTCGATGTTAACCA-3′ (upper primer) and 5′-TTCAATCCCCAAATCCTAGCC-3′ (lower primer). The PCR reactions were performed as follows: 94°C for 4 min; 35 cycles at 95°C for 30 s, 57°C for 40 s, and 72°C for 40 s; 72°C for 10 min.

### Reagents

The rabbit anti-hTNFα polyclonal antibody for immunohistochemistry were purchased from Abcam (no. ab9635, Cambridge, UK). AT132, a preclinical human monoclonal antibody against TNFα, was provided by Livzon MabPharm Inc. (Zhuhai, China). Adalimumab, a human monoclonal antibody against TNFα was purchased from AbbVie Inc. (North Chicago, USA). Infliximab, a human/mouse chimeric monoclonal antibody against TNFα was purchased from Janssen Biotech, Inc (Malvern, USA). BD Cytometric Bead Array Human TNF kit was purchased from BD Biosciences Inc. (San Jose, USA). Rabbit horseradish peroxidase kit for immunohistochemistry was purchased from CWBiotech Inc. (Beijing, China).

### Clinical assessment

Weekly body weight and arthritis scores in all four limbs were recorded after weaning (3 weeks of age). Clinical severity of arthritis for each paw (fingers, tarsus, and ankle) was quantified by attributing a score ranging from 0 to 3: 0, normal; 1, slight redness and/or swelling; 2, pronounced edematous swelling; 3, joint deformity and rigidity (Williams et al., 1992). The arthritis score per mouse was an average of the four limbs.

### Histology

Peripheral joints were removed from animals and were fixed in 10% buffered formalin for two days, flushed by deionized water overnight, and then decalcified in 30% formic acid for 4 days, finally embedded in paraffin. Other tissues were subjected to the same procedure except for decalcification. Sections of 3 μm were cut and stained with haematoxylin and eosin according to standard procedures, then evaluated microscopically.

### Immunohistochemistry

After dewaxing, rehydration, and high-temperature antigen retrieval with 0.01% sodium citrate buffer (pH 6.0), the sections were immunostained with rabbit anti-hTNFα antibody (1:200). Subsequently, they were incubated with biotinylated goat anti-rabbit IgG (1:200) and avidin-biotin-peroxidase complex. Peroxidase activity was detected using diaminobenzidine for 1 min. Finally, the sections were counterstained with hematoxylin. Non-immune rabbit serum was used as a control.

### Cytometric bead array

The synovial membranes of bilateral knee joints were excised from the same mice. The tissues were weighed and homogenated in 100 μl pre-cooled PBS, then the suspension was centrifuged to obtain the tissue supernatant.

The assay was followed by the commercially available cytometric bead array platform protocol. Briefly, microbeads with fluorescence signals were coated with capture antibodies specific for hTNFα.50 μl of the capture bead, phycoerythrin-conjugated detection antibodies, was mixed with 50 μl of the samples or recombinant standards used to generate a standard curve, vortexed, and then incubated for 2.5 h at room temperature. Samples were washed by the addition of 1 ml of wash buffer and then centrifuged at 200× ***g*** for 5 min. The supernatant was aspirated, and then 50 μl of phycoerythrin detection reagent was added to each tube, after vortexing, incubated the samples for 30 min at room temperature. Samples were then washed once as described above, resuspended in 300 μl of wash buffer. Before the CBA was started, the cytometer setup beads provided in the kit were used for the photomultiplier tube voltage and compensation settings in the BD FACSCanto™ II flow cytometer (Becton Dickinson, Franklin Lakes, USA). The singlet bead population was obtained by adjustment of the forward and side light scatter voltages. The samples were analyzed on the cytometer using BD CBA analysis software (Becton Dickinson, Franklin Lakes, USA). The concentration of hTNFα in pg/g was determined based on a 10-point standard curve generated using the recombinant standards provided in the kit.

### Evaluation of hTNFα monoclonal antibodies

According to the weight, from the age of 3 to 10 weeks, TgTC mice received one time weekly intraperitoneal injections of anti-hTNFα monoclonal antibody (AT132 at 1.25, 0.625, 0.312 mg/kg; Adalimumab at 1.2, 0.6, 0.3 mg/kg; Infliximab at 60, 30, 15 mg/kg) dissolved in saline, while the control intraperitoneally injected the saline. More than five of TgTC mice were used in each group. The clinical scores were recorded in both monoclonal antibody and saline treated groups every week, and all groups were sacrificed for histopathological and hTNFα quantitative analysis at 10 weeks of age.

### Statistical analysis

Statistical significance between two groups was evaluated by a two-tailed unpaired *t*-test with Welch's correction, using GraphPad Prism 6.01 software version for Windows (San Diego, CA, USA). Data were shown as mean±s.e.m. Data shown is from a representative experiment of three independent experiments, unless otherwise noted. Statistical significance was defined as *P<0.05, **P<0.01, ***P<0.001 and ****P<0.0001.
